# Stent graft placement and balloon dilation for pseudoaneurysm complicated by distal arterial stenosis of the hepatic artery after pancreaticoduodenectomy

**DOI:** 10.1186/s40792-015-0060-2

**Published:** 2015-07-22

**Authors:** Shuichi Fujioka, Fumitake Suzuki, Naotake Funamizu, Tomoyoshi Okamoto, Koji Munakata, Hirokazu Ashida, Katsuhiko Yanaga

**Affiliations:** Department of Surgery, The Jikei University Daisan Hospital, 4-11-1, Izumi-honcho, Komae City, 201-0003 Tokyo Japan; Department of Radiology, The Jikei Daisan Hospital, Tokyo, 201-0003 Japan; Department of Surgery, The Jikei University School of Medicine, Tokyo, 105-8461 Japan

**Keywords:** Pseudoaneurysm, Interventional radiology, Stent graft, Balloon dilation

## Abstract

Hemorrhage from ruptured pseudoaneurysm is a rapidly progressing and potentially fatal complication after pancreaticoduodenectomy (PD). Stent graft placement for hepatic artery pseudoaneurysm has recently been reported as a valid alternative to transcatheter arterial embolization (TAE). We report a case of pseudoaneurysm of the common hepatic artery (CHA) with distal arterial stenosis treated by stent graft placement for pseudoaneurysm and balloon dilation for arterial stenosis due to pancreatic fistula after PD. A 67-year-old man underwent PD for intraductal papillary mucinous neoplasm with concomitant early gastric cancer. After the operation, pancreatic fistula developed, for which conservative management by drainage was continued. On the postoperative day 30, melena started. Emergency abdominal angiography revealed a pseudoaneurysm in the CHA, as well as distal arterial stenosis extending from the proper hepatic artery (PHA) to bilateral hepatic arteries. The portal vein was also stenotic due to pancreatic fistula, for which TAE was not judged suitable because of the risk of liver failure. Therefore, stent graft placement and balloon dilation were chosen. Three pieces of coronary covered stent were placed in a coaxial overlapping manner followed by balloon dilation of the proper and left hepatic arteries. Balloon dilation of the right hepatic artery failed by technical reasons. Completion arteriography confirmed the patency from the CHA to the left hepatic artery as well as the exclusion of the pseudoaneurysm. A liver abscess that developed in the right hepatic lobe after intervention was successfully treated by percutaneous drainage, and the patient discharged on day 27 after stent graft placement. Non-embolic management with preservation of the liver arterial flow may be an option for complicated pseudoaneurysm after PD.

## Background

Despite the advancement of surgical techniques, post-pancreatectomy hemorrhage remains one of the most serious complications, occurring in 1–8 % of all pancreatic resections and accounting for 11–38 % of overall mortalities [1–3]. Among them, delayed hemorrhage after pancreaticoduodenectomy (PD) is defined as bleeding manifesting over 24 h [3]. Delayed hemorrhage is commonly attributable to pseudoaneurysms of the common hepatic artery (CHA) or superior mesenteric artery, which were induced by postoperative pancreatic fistula (POPF). Conventional management involves surgical re-exploration or transcatheter arterial embolization (TAE) of the bleeding vessels [4]. Recently, as an alternative treatment option, use of a coronary covered stent to exclude pseudoaneurysms has been reported sporadically [5–7]. The potential of stent graft placement as initial treatment of hemorrhage from pseudoaneurysms has slowly been recognized. While application criteria of stent graft placement are yet to be established, the arterial pseudoaneurysm attributable to POPF should be treated carefully. The management of pseudoaneurysm should be decided considering bloodstream to the liver as well as the size and the fragility of the pseudoaneurysm. POPF-induced pseudoaneurysms are usually fragile because of severe local inflammation with infection that weakens the arterial wall. We report a case of CHA pseudoaneurysm complicated by distal hepatic artery stenosis secondary to POPF after PD, which was treated with coronary stent grafts and balloon dilation.

## Case presentation

**Fig. 1:**
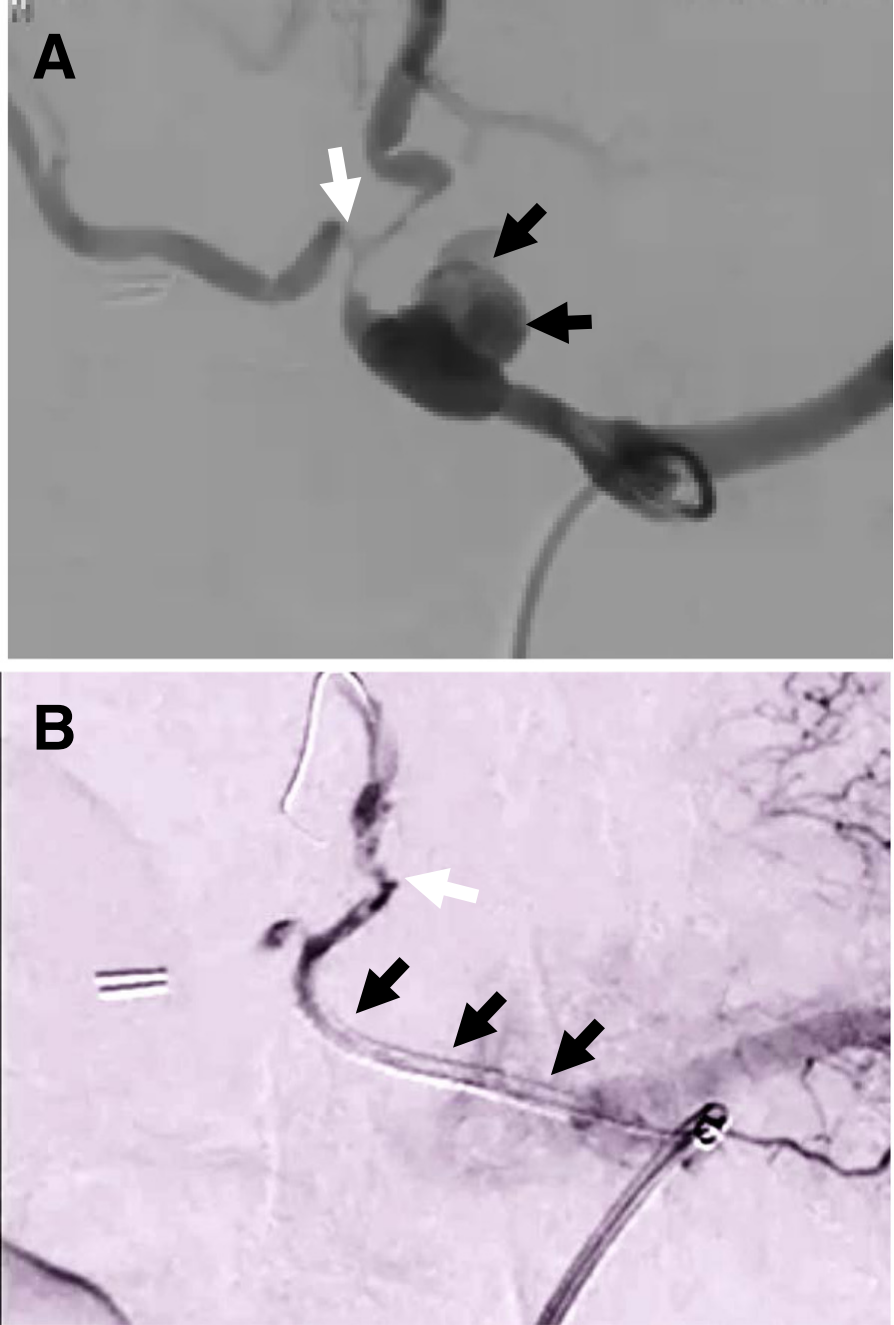
**a** Emergency celiac arteriography revealed a common hepatic artery pseudoaneurysm (*black arrows*). The distal side of the arterial lumen was markedly stenotic (*white arrow*). **b** Three pieces of coronary covered stent (*black arrows*), 3.5 mm in diameter and 16 mm in length, were placed in the common hepatic artery. The proper and left hepatic arteries (*white arrow*) were dilated by a balloon catheter

**Fig. 2:**
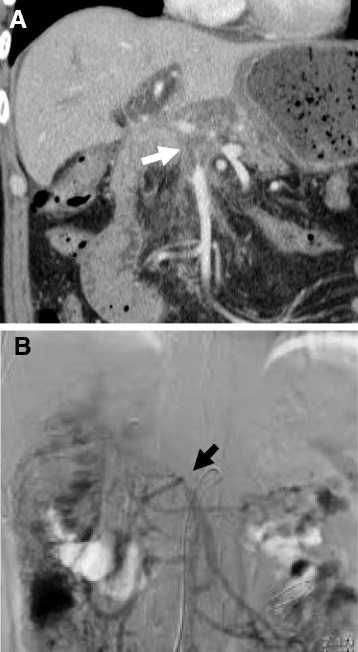
Portal phase of emergency CT (**a**) and angiography (**b**). A grossly stenotic portal vein due to postoperative pancreatic fistula was confirmed (white and black arrow)

**Fig. 3:**
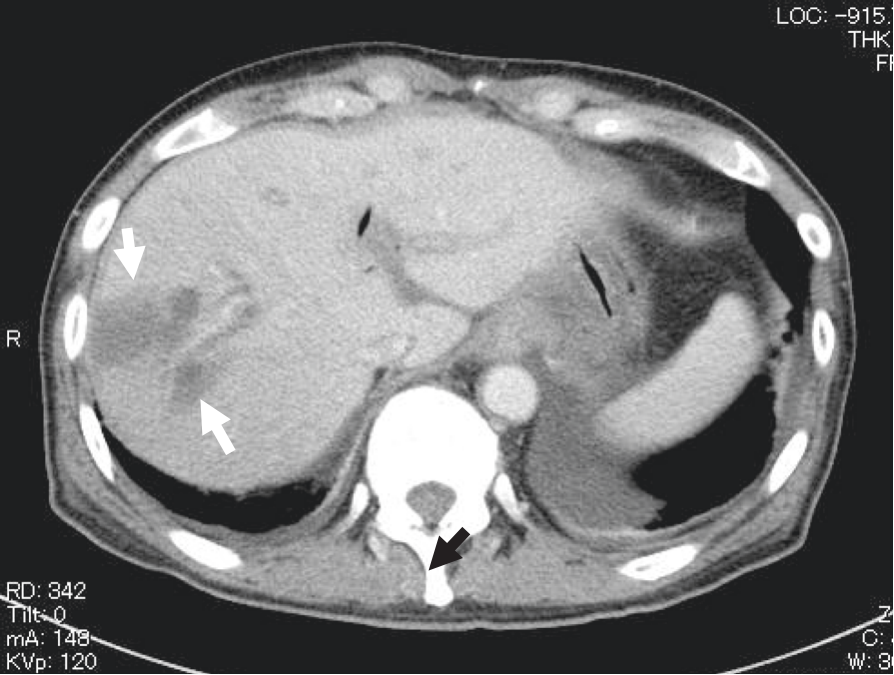
CT image which has been performed 14 days after stent graft placement. A low-density area corresponding to the liver abscess was observed in the right hepatic lobe

**Fig. 4:**
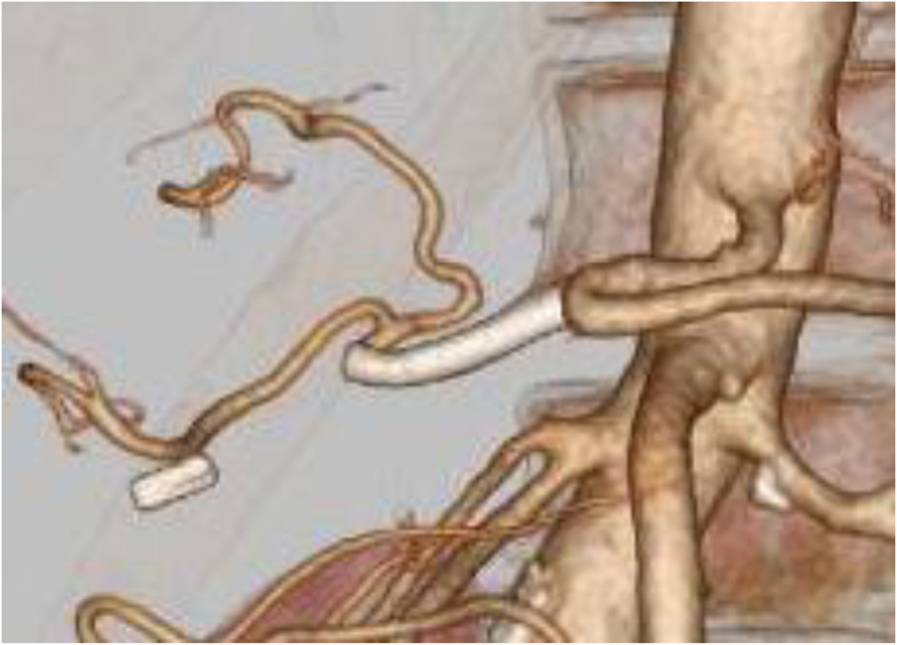
CT arteriography performed 10 months after stent graft placement, which indicated the patency of the stent graft without stenosis of distal hepatic arteries

**Fig. 5:**
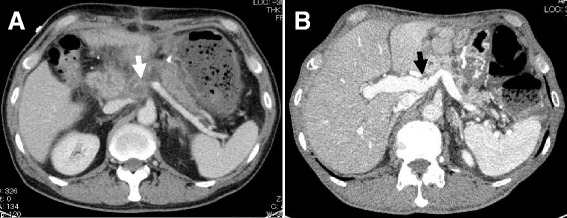
A comparison of the portal vein stenosis assessed by CT images of the portal phase between right before (**a**) and 10 months after (**b**) stent graft placement. Portal vein stenosis (**a**, *white arrow*) caused by postoperative pancreatic fistula was significantly improved (**b**, *black arrow*)

A 67-year-old man underwent PD for intraductal papillary mucinous neoplasm of the pancreas head with concomitant early gastric cancer originating from the gastric angle. The gastric lesion was diagnosed as signet ring cell carcinoma, which was suspected of cancer invasion to the submucosal layer. Distal gastrectomy and lymph node dissection with left gastric artery isolation were added for gastric cancer. Reconstruction procedure was performed with the modified Child method, comprising pancreaticojejunostomy, choledochojejunostomy, and gastrojejunostomy. End-to-side pancreaticojejunostomy was accomplished by duct-to-mucosa anastomosis (modified Kakita method) with monofilament absorbable thread. An external stent across the pancreaticojejunostomy has been placed for diversion of pancreatic juice from the pancreatic anastomotic site. A closed suction drain was placed to the ventral part of the pancreaticojejunostomy. On postoperative day (POD) 5, a turbid discharge was observed from the drainage. The drainage juice contained amylase of more than 10,000 IU/ml, which was confirmed as pancreatic fistula based on the criteria of the International Study Group on Pancreatic Fistula [8], for which continuous suction drainage was initiated. The pancreatic duct drainage tube was removed on POD 21 because of obstruction. Since then, drainage characteristics changed to contain digestive juice, which indicated that the bile and the pancreatic juice were mixed and leaked out from the pancreaticojejunostomy. The patient showed melena without circulatory disturbance on POD 30. Emergency angiography was performed, which revealed a CHA pseudoaneurysm with distal stenosis of the proper and bilateral hepatic arteries (Fig. 1a). No extravasation was apparent. These findings were consistent with the leakage of the pancreaticojejunostomy, through which gastrointestinal hemorrhage from the pseudoaneurysm developed. To make matters worse, the CHA pseudoaneurysm was complicated by portal vein stenosis which could cause post-TAE complications such as liver failure (Fig. 2). Therefore, coronary covered stenting was chosen to treat the pseudoaneurysm and to maintain hepatic arterial flow, instead of performing conventional TAE. The diameter of the CHA was measured under subtraction angiography, and three pieces of the JOSTENT GraftMaster (Abbott Vascular, Redwood City, CA, USA), 3.5 mm in diameter and 16 mm in length, were placed in the CHA through a guidewire. Subsequently, the proper and the left hepatic arteries were dilated using a balloon dilator of 4.5 mm. The balloon dilation of the right hepatic artery was unsuccessful for technical reasons. After all, angiography confirmed exclusion of the CHA pseudoaneurysm and maintenance of arterial blood flow of the liver (Fig. 1b). No vascular events such as dissection, thromboembolic occlusions, or any vascular damage to the celiac axis or hepatic arteries were encountered during or after the procedure. His blood pressure remained stable, and the cessation of gastrointestinal hemorrhage was confirmed. The peak transaminase levels rose up to AST of 723 U/ml and ALT of 1136 IU/ml on day 1 after the procedure. Liver abscess developed in the right hepatic robe on CT scans on day 14 (Fig. 3), which was treated by percutaneous drainage. POPF ceased after stent graft placement spontaneously, and the external drainage tube was removed the next day after stent graft placement. He was discharged POD 57, 27 days after stent graft placement, and remained well. CT arteriography which has been performed 10 months after stent placement indicated the patency of stent grafts without stenosis of the distal hepatic artery as well as the improvement of the portal vein stenosis (Figs. 4 and 5).

### Discussion

TAE and stent graft placement for delayed hemorrhage from arterial pseudoaneurysm after PD represent a first-line treatment, due to their high success and low morbidity rates as compared with surgical re-laparotomy [9]. Treatment selection of endovascular intervention between TAE and stent graft placement is usually given individually depending on the involved artery and its vascular anatomy. Generally, the liver can tolerate CHA embolization without major complications because of collaterals which are supplied from left hepatic or inferior phrenic arteries [10]. However, TAE of the CHA may cause fatal hepatic necrosis after PD in the absence of collateral arteries, as previously described [1]. Therefore, in cases in which TAE of the hepatic artery is planned for the CHA pseudoaneurysm, the presence of adequate blood flow of the portal vein is essential [11]. Thus, stent graft placement would be preferable under conditions where either the portal vein is occluded by POPF or collateral arterial blood flow is not expected. Vital signs may be insignificant for the selection of TAE or stent graft. In the present case, stent graft was chosen to exclude the pseudoaneurysm because the blood flow of the portal vein was markedly decreased. Collateral arterial flow from the left gastric artery could not be expected by lymphadenectomy for gastric cancer. Moreover, the distal part of the CHA was constricted presumably due to local inflammation by POPF. In order to manage these complicated situations, stent graft placement of the CHA to exclude the pseudoaneurysm and balloon dilation of the distal arterial stenosis were chosen. Interventional balloon dilation for right hepatic artery failed, through which liver abscess may appear in the right liver robe after the procedure. By using stent graft placement and distal arterial balloon dilation, the left hepatic artery was successfully preserved, which seems important to avoid liver failure. Stent graft patency was confirmed by CT arteriography which was taken 10 months after placement. Moreover, significant improvement of both hepatic artery and portal vein stenosis was observed, suggesting these pathological findings were a temporary condition as a result of inflammatory vascular erosion related to POPF. Thus, the acute phase of pseudoaneurysm formation after POPF can reveal various and complicated findings which can affect the bloodstream to the liver. Under these circumstances, TAE for the CHA may be attributable to liver failure. Theoretically, stent graft insertion for the exclusion of pseudoaneurysm and balloon dilation for arteriostenosis are useful as the initial treatment for CHA pseudoaneurysm in order to manage transient and acute hepatic blood reduction caused by POPF. In 2014, Asai et al. reviewed 11 case reports and 4 small case series published in the English literature [7]. Among 34 cases of stent graft placement for the treatment of bleeding pseudoaneurysm, 22 cases originated from the CHA. The JOSTENT GraftMaster was the most commonly used stent graft in recent series and the current case, which is indicated only for perforation of the coronary arteries [12]. The Jostent is small and short as compared with conventional covered stents and is suitable for making placement through the celiac artery. Post-placement stent graft infection may be theoretically possible, because the stent graft material may be colonized and infected. Local infection by POPF cannot be usually controlled perfectly. Asai et al. indicated that among the 22 patients with CHA pseudoaneurysm due to POPF, three deaths were attributed to septicemia after the placement of a stent graft [7]. The rate of infection between TAE and stent graft placement may be considered equivalent, in terms of artificial material placement inside the artery [13, 14]. In the present case, melena has been observed, which represents rupture of the CHA pseudoaneurysm, which in turn suggests the existence of a communication between the pseudoaneurysm and the digestive tract. However, we have chosen stent graft placement instead of TAE to exclude the CHA pseudoaneurysm because a potential contamination with bacteria is unavoidable in either management. POPF spontaneously ceased after pseudoaneurysm rupture. Indeed, the drain discharge stopped after stent graft placement and the external drain could be removed. A possible explanation of this clinical progress may be due to the formation of an internal fistula caused by pseudoaneurysm rupture and following increase of local pressure. Ding et al. reported significant low mortality of endovascular intervention including stent graft placement and TAE as compared with surgical treatment for ruptured visceral artery pseudoaneurysms in patients who underwent pancreatic surgery [9]. These reports suggest that endovascular intervention was considered to be superior to surgical management in spite of the potential of post-placement infection conclusively.

## Conclusions

The arterial pseudoaneurysm caused by POPF after PD seems to require careful evaluation according to clinical conditions such as the complexity, fragility, and shape of the pseudoaneurysm as well as the bloodstream to the liver. Stent graft placement for a bleeding pseudoaneurysm after PD may replace TAE as the first-line treatment, under selected conditions.

## Consent

Written informed consent was obtained from the patient for publication of this case report and any accompanying images. A copy of the written consent is available for review by the Editor-in-Chief of this journal.

## Abbreviations

PD: Pancreaticoduodenectomy
TAE: Transcatheter arterial embolization
CHA: Common hepatic artery
PHA: Proper hepatic artery
POPF: Postoperative pancreatic fistula
POD: Postoperative day
